# Cultivating key disciplinary competencies among university students against the background of China’s new engineering education initiative

**DOI:** 10.1371/journal.pone.0318561

**Published:** 2025-02-14

**Authors:** Liyuan Zheng, Di Ge

**Affiliations:** School of Information and Intelligence, Zhejiang Wanli University, Ningbo, Zhejiang, China; Universiti Sains Malaysia - Kampus Kesihatan, MALAYSIA

## Abstract

The development of students’ core disciplinary competencies is crucial to the quality of talent cultivation against the background of China’s New Engineering Education initiative. We condensed a literacy–ability–knowledge model of the core competence structure of university students based on the results of a survey and then applied this model with a special focus on competence structures. We coded the core competency indicators of the course area using a software engineering course as an example to construct a map of the core competencies of the course (area). New teaching and instruction methods to promote the development of students’ core competencies were then explored. Finally, to shape core subject competence, we suggest building a course recommendation system that aligns with an employment guidance service based on the certification of core subject competence to realize a new mode of cultivating competence.

## 1. Introduction

Since joining the Washington Accord in 2016, China has proposed a New Engineering Education (NEE) initiative to reform its university-level engineering education. NEE includes the cross-fertilization of disciplines, the combination of science and engineering, and the penetration of engineering and literature, which breeds cross-specialization and an educational model that cultivates engineering talents across faculties, disciplines, and specialties [1]. This kind of education must be student-centered and focused on cultivating students’ ability to think independently, innovate, and apply their knowledge in practice while also including an international vision and sense of social responsibility. It also requires stronger cooperation among industry and universities, as well as the promotion of a close integration of engineering education with scientific and technological innovation, alongside economic and social development [2].

The shift from traditional engineering education to the NEE approach is a response to the imperatives of the scientific and technological revolution and industrial transformation of modern society. The cultivation of new engineering talents is challenging. The dynamic interplay of labor supply and demand requires a nuanced understanding of the alignment between students’ demands for skills and the responsiveness of universities to societal needs. Within the student-centric education model, however, effective technological scrutiny of the nexus of students’ literacy, abilities, and knowledge is lacking [3]. The acquisition of this knowledge would furnish decision-makers with substantive data to support the formulation of innovative teaching models [4].

Given these challenges, this study considers the requirements for cultivating university students’ key disciplinary competencies in the context of NEE; relevant courses are used as examples to construct a map of these competencies in the course area. We also realized real-time competency diagnosis of a specific group of students by building a portrait of key disciplinary competencies to provide personalized development programs. We also explored a new mode of instruction to promote the development of student competence from a teaching and learning perspective. This paper makes the following primary innovations and contributions:

1. Proposal of a structural model for the key disciplinary competencies of university students that incorporates three evaluative indices: literacy, ability, and knowledge (LAK). We therefore call it the LAK model, and it provides a guiding framework for the formulation of curriculum implementation standards and the exploration of ways to cultivate talent.
2. Exploration of the practical application of the LAK model, including the encoding of key competence indicators and the creation of maps or portraits of these competencies. This provides novel perspectives for exploring innovative methods of teaching, thus furthering students’ pivotal disciplinary competencies.
3. We suggest countermeasures to shape college students’ competencies based on case studies to improve their professional development

## 2. Related work

The swift progression of information technology and the pervasive influence of artificial intelligence across various domains have given rise to a multitude of interdisciplinary industries in recent years [5]. This transformation has placed higher education at a crucial historical juncture that emphasizes the prevailing trend of multidisciplinary integration and the need to cultivate composite innovative talents [6]. Higher engineering education must expedite the nurturing of versatile and innovative new engineering talents, particularly in anticipation of the burgeoning future industries, industrial amalgamation, the trajectory of the new economy, and the evolving landscape of new business developments, coupled with intensified global competition in these spheres [7]. This has heralded a new chapter in the establishment and advancement of new engineering on a global scale, which requires the adoption of new methods and approaches in the paradigm for cultivating engineering talent.

Supporting competence in key disciplines among university students has emerged as a focal point in educational research, which has been subjected to numerous comprehensive investigations. According to Mojarradi and Karamidehkordi [8], disciplinary competence is a psychological regulatory mechanism that facilitates the smooth execution of corresponding disciplinary activities. Understanding the factors that influence students’ levels of disciplinary competence has significant value for curriculum design and instruction. In their meticulous analysis of the intrinsic structure of disciplinary competence, Moliner et al. [9] introduced the components of disciplinary knowledge, real-life situations, and evaluation criteria of disciplinary competence; this analysis resulted in the formulation of a model integrating these essential elements. Gutierrez-Bucheli et al. [10] investigated the distinctions among different disciplinary competence structures and asserted that the disciplinary competence model is a pivotal link for establishing standards of educational quality and implementing educational goals.

In the United States, it has been suggested that merely assessing test scores related to knowledge and skills is insufficient for predicting graduates’ success in their future careers. Core competencies are crucial for students’ successful employment and sustainable development [11,12]. Globally, the higher education community is increasingly endorsing competency-based education, with a shift in focus from knowledge and skills to the cultivation and assessment of core competencies. This approach seeks to ensure graduates meet the demand for high-quality talents from enterprises and society [13]. The U.S. Bureau of Labor Statistics (Employment and Training) introduced a generic competency model structured as a nine-layer pyramid. The foundational layer comprises generic competencies, and the pyramid progresses upward to represent more specific occupational competencies. The layers include basic competencies (Layers 1–3), industry-related competencies (Layers 4 and 5), and occupation-related competencies (Layers 6–9), which encompass knowledge, skills, requirements, and managerial competencies [14]. A methodology for forecasting competence needs based on technology roadmap analysis has been proposed, which has contributed to the understanding of the competency needs of Industry 4.0. This qualitative analysis of the competence structure required by Industry 4.0 provides insights into the evolving landscape of knowledge, skills, and attitudes required in this technological era.

The cultivation of university students’ competence in key disciplines has immense significance for curriculum design, teaching methodologies, and educational quality standards. It not only provides valuable insights into the exploration of modes for cultivating talent within the NEE framework but is also a methodological and path-guiding resource. This study therefore positions the cultivation of university students’ key disciplinary competence as its overarching objective and explores the mechanisms for nurturing and enhancing such competence within the new engineering context to offer a practical blueprint for instruction in this field through curriculum development.

## 3. NEE survey and construction of the LAK model

In response to the pragmatic requirements for training new engineers, we investigated the needed competencies and instruction strategies for information-based new engineering talents among college educators and industry stakeholders. The primary research methods used were questionnaires. A questionnaire rated on a 5-point Likert scale was tailored to the curriculum characteristics of information disciplines to delineate the required competencies across 25 distinctive elements. Survey participants were then prompted to evaluate the importance of these elements. The questionnaires were distributed online to 90 respondents (comprising college teachers and HR representatives from enterprises), and a total of 85 filled-in questionnaires were collected. Of these, 80 were deemed valid (see Table 1 for specific parameters).

**Table 1:** Questionnaire survey sample composition.

Category	Composition of survey samples		
**College teachers**	**Enterprise personnel**	**Total**
Total number of samples	45	45	90
Recycled samples	45	40	85
Valid samples	43	37	80

**Note:** Only fully completed samples (i.e., recycled samples) were collected; the total valid samples were obtained after excluding samples with a short completion time.

To ensure the validity of the questionnaire content, items were validated through a reliability test using SPSS 20.0. The questionnaire’s Cronbach’s alpha coefficient was 0.811 (greater than 0.8), which indicates good overall reliability [15]. With a KMO value of 0.785 (greater than 0.7), the results of Bartlett’s test of sphericity also showed that the questionnaire was significant at the 0.001 level and that the factor loading coefficients met the minimum requirements, thus indicating that the questionnaire possesses good validity [16,17].

The different survey respondents had different ideas about which competencies information-based new engineering talents should have, so the means and standard deviations of the evaluation scores for each competence were counted, as shown in Table 2.

**Table 2:** Statistics on the scoring of capacity needs.

Category	Needed abilities	College teachers		Enterprise personnel	
**Mean**	**Standard deviation**	**Mean**	**Standard deviation**
**Knowledge**	Professional fundamentals	4.15	0.46	4.05	0.57
	Engineering fundamentals	4.35	0.55	4.51	0.61
	Information technology	4.06	0.64	4.18	0.62
	Artificial intelligence	4.20	0.65	4.27	0.60
	Interdisciplinarity	3.86	0.67	4.43	0.50
	Environmental awareness	4.35	0.48	3.78	0.48
	Legal competency	3.95	0.46	4.16	0.50
**0Ability**	Information retrieval	4.46	0.50	4.19	0.50
	Use of new media tools	4.02	0.53	4.18	0.46
	Interdisciplinary learning	4.24	0.53	4.18	0.51
	Data analysis	4.37	0.48	4.41	0.49
	Data maintenance	3.49	0.87	4.02	0.72
	Data decision-making	3.80	0.54	3.97	0.68
	Computer programming	4.31	0.54	4.62	0.49
	Human–computer interaction	3.69	0.49	3.94	0.46
	Problem solving	4.57	0.51	4.43	0.50
	Teamwork	4.53	0.51	4.49	0.51
	Academic writing	3.96	0.46	4.16	0.55
	Lifelong learning	4.29	0.42	4.28	0.47
	Adaptability to change	4.16	0.52	4.24	0.54
**Literacy**	Professional literacy	4.50	0.49	4.48	0.51
	Dedication	4.37	0.48	4.54	0.51
	Innovative ideas	4.42	0.50	4.46	0.50
	Steadfastness	4.11	0.55	4.37	0.55
	Humanistic	4.60	0.50	4.54	0.51

**Note:** Scores ranged from 1 to 5; higher scores represent stronger needs.

**Fig 1:**
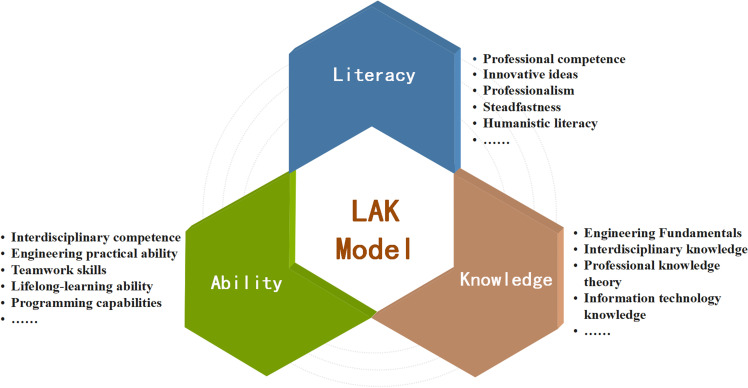
Literacy–ability–knowledge model of key disciplinary competencies.

Factor analysis was used to categorize the shared variables within the original variables of demanded abilities for information-oriented new engineering talents to extract robust explanatory factors. The subsequent analysis, based on the rotation component matrix results, yielded three primary ability components: literacy, ability, and knowledge. These components provide a nuanced understanding of the required competencies of the target talent group. Building upon the survey and analysis outcomes, the focus was narrowed to the key competencies across these three dimensions to create a comprehensive model of the key disciplinary competency structure. This LAK model was formulated for university students within the realm of new engineering, as shown in Fig 1, and it provides a guiding framework for shaping curriculum implementation standards and exploring methodologies for cultivating innovative talents.

## 4. Application of the LAK model

Leveraging its comprehensive rich competence evaluation framework, the LAK model provides a supportive framework for shaping a professional curriculum system and quantifying indices of competence within the curriculum. Using the model as a benchmark, a multi-dimensional competence certification coding structure and visualization map for an individual course or curriculum system can be developed to facilitate the seamless implementation of effective instruction. Software engineering is a core specialty of the new engineering discipline, so it was used as an example, and the application of the model is described in detail in the following text.

### 4.1. Coding indicators

The LAK model is used as a research paradigm. The core specialized information-class courses in the context of new engineering (e.g., software engineering, communication principles and applications, digital image processing, machine learning, and natural language processing) were selected for coding study disciplinary competence indicators in the curriculum system. An n ×  3 key disciplinary competency analysis system was introduced for each course, as exemplified in Table 3. The key disciplinary competencies for each specialized course were categorized into three levels—literacy (L), ability (A), and knowledge (K)—based on the proposed LAK model. Specific competencies are outlined under each dimension to create a framework for a competence indicator system specific to each course. Example indicators include professionalism (L1), dedication (L2), and innovative ideas (L3) for literacy; personal efficacy (A1), academic competence (A2), social competence (A3), and technological competence (A4) for ability; and integration of basic knowledge from related curriculum (K1), intermediate-order knowledge (K2), and higher-order knowledge (K3) for knowledge.

**Table 3:** Demonstrated n ×  3 system for a course’s key disciplinary competencies.

Course name	Dimensions					
	Literacy (L)		Ability (A)		Knowledge (K)	
Software engineering	L1 Professionalism	L1-1	A1 Personal effectiveness	A1-1	K1 Basics	K1-1
		L1-2		A1-2		K1-2
		…		…		…
	L2 Dedication	L2-1	A2 Academic ability	A2-1	K2 Intermediate knowledge	K2-1
		L2-2		A2-2		K2-2
		…		…		…
	L3 Innovative ideas	L3-1	A3 Social competence	A3-1	K3 Advanced knowledge	K3-1
		L3-2		A3-2		K3-2
		…		…		…
	L4 Steadfastness	L4-1	A4 Technical capacity	A4-1	*	*
		L4-2		A4-2		
		…		…		
	L5 Humanistic literacy	L5-1	*	*	*	*
		L5-2				
		…				
Other courses	…	…	…	…	…	…

Based on this n ×  3 system and the required competencies for the software engineering course, the specific key indices were expanded based on the instructional requirements to form a multi-level, three-dimensional reference standard for key disciplinary competencies (for the specific coding, see Table 4).

**Table 4:** Coding sample: Key disciplinary competencies for software engineering. (* Indicates additional indicators that can be expanded).

Structure of key disciplinary competencies for software engineering	Level 1	Level 2	Definition
Literacy (L)–SE	L1 Professional literacy	L1-1 (abstract thinking)	Able to abstractly think about relevant things
		L1-2 (critical thinking)	Able to evaluate and reflect on certain standards
	L2 Professionalism	L2-1 (professional ideal)	Able to establish firm career ideals and have a strong sense of career ambition
		L2-2 (purpose of job)	Able to focus on one’s career, down-to-earth, diligent, and responsible
	L3 Innovative ideas	L3-1 (global concept)	Able to integrate one’s own development into the global development landscape
		L3-2 (exploratory spirit)	Able to use one’s own knowledge to explore new fields
	L4 Steadfastness	L4-1 (passion)	Able to maintain sustained passion in pursuit of long-term goals
		L4-2 (persistence)	The tendency to be resilient, responsible, and hardworking
	L5 Humanistic literacy	L5-1 (humanistic knowledge)	Knowledge of history, politics, law, and philosophy, among other humanistic topics
		L5-2 (humanistic spirit)	Able to pursue a better state of life and society
	L	…	…
Ability (A)–SE	A1 Personal effectiveness	A1-1 (lifelong learning)	Able to maintain a lifelong learning attitude
		A1-2 (self-management)	Able to carry out effective self-discipline and management
	A2 Academic aptitude	A2-1 (academic writing)	Able to write papers on the research direction
		A2-2 (information literacy)	Able to use tools for data retrieval
	A3 Social ability	A3-1 (expressing communication)	Able to communicate effectively with others
		A3-2 (solidarity)	Capable of task division and collaboration
	A4 Technical ability	A4-1 (programming)	Able to conduct theoretical analysis of data
		A4-2 (program analysis)	Able to use information technology to solve problems
	A^*^	…	…
Knowledge (K)–SE	K1 Basic knowledge	K1-1 (requirement analysis)	Able to master the basic knowledge of demand analysis
		K1-2 (software design)	Able to design network software
		K1-3 (software maintenance)	Basic maintenance of design software
	K2 Intermediate knowledge	K2-1 (modular design)	Capable of modular design of a network system
	K3 Advanced knowledge	K3-1 (data security)	Able to ensure the data security of the development system
		K3-2 (project programming)	Capable of project programming
	K^*^	…	…

### 4.2. Constructing a portrait of core subject competence

Drawing on the coding specifications from Table 4, learning analytics and data mining were used to craft a multidisciplinary profile of key disciplinary competencies among engineering students to target those competencies in specific engineering student groups. The generated portrait, tailored to the specific characteristics of each group, offers visually personalized competency labels and tailored program recommendations. This personalized approach may motivate students to enhance their individual or group-specific key disciplinary competencies proactively, thereby advancing teaching quality and the relevance of instruction.

#### 4.2.1. Subject ability portrait based on instruction.

**Fig 2:**
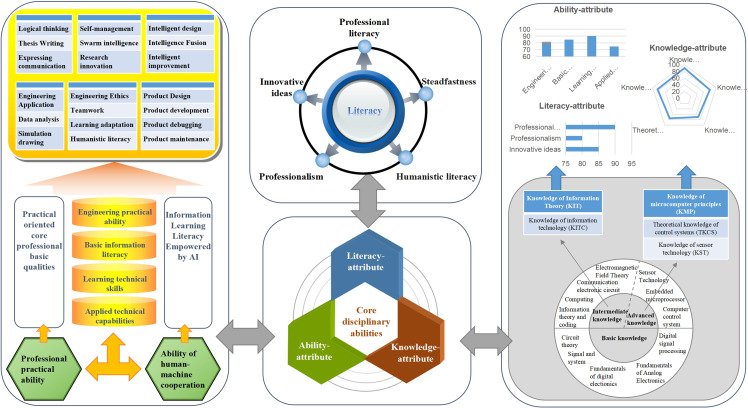
Portrait of software engineering students’ key subject competencies.

Digital portrait technology using artificial intelligence and big data is effective for real-time analysis and accurate portrayal of the values for students’ ability state [18]. The structural LAK model was therefore used as the core element to construct the core disciplinary competency portraits of software engineering students in the context of the NEE initiative with the help of techniques such as data mining and visualization and analysis, as shown in Fig 2.

The competence portrait for software engineering students classified the knowledge attribute into five distinct categories: information theory, information technology, microcomputer principles, theory of control systems, and sensor technology; it is primarily visualized on the learning platform through course performance. Given the practice-oriented foundational professional qualities and information literacy prerequisites, key components of the ability attribute were identified as professional practice and human–computer collaboration. The literacy attribute was also grouped into five categories to create a comprehensive portrayal indicating multidimensional literacy: professionalism, dedication, innovation, perseverance, and humanistic literacy. The resulting portrait is a valuable tool for self-diagnosis and evaluating learning performance for the target population, as well as proving an effective reference point for advancing their cognitive development.

#### 4.2.2. Disciplinary portrait based on competitions.

**Fig 3:**
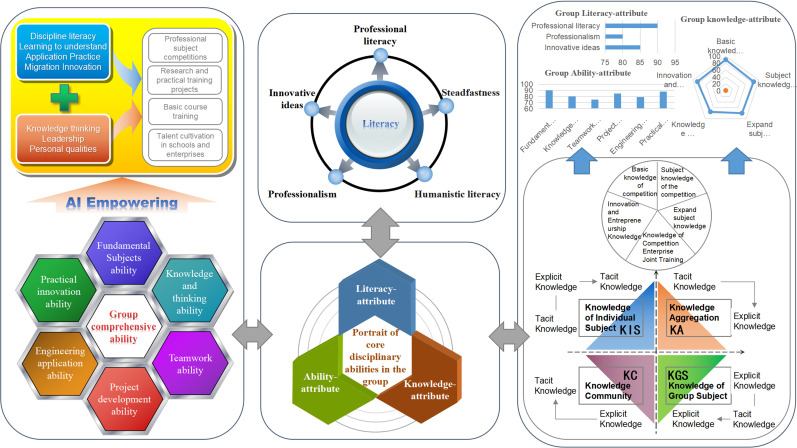
Portrait of clustered key disciplinary competencies.

The individual key disciplinary competence portrait provides a characterization tailored for single-person tasks, but its applicability diminishes for multi-person tasks, such as participating in disciplinary competitions within teams or group practical training. These limitations were addressed by constructing a competition-type competence portrait for groups. The competition among new engineering majors is taken as an illustrative example and depicted in Fig 3.

The group portrait identifies various elements that are crucial for group members, encompassing the three dimensions of the LAK model within the specific context of disciplinary competitions. This comprehensive perspective can help group members in decision-making by providing insights into their behavioral performance, strengths, weaknesses, and existing challenges across multiple dimensions. These group portraits scale systematically, and the integrated visualized development report derived from the group portraits of participating members dynamically presents the overall status of disciplinary competence for the entire team in the competition. This facilitates the monitoring and management of the competition schedule by decision-makers, thus enabling the formulation of plans to enhance the team’s performance.

Multi-disciplinary student competence profiles serve a dual purpose: They help learners engage in self-examination and reflect on their developmental constraints, including their knowledge, abilities, and innovation quality, and they also offer valuable data to support teachers in assessing student learning and for school administrators to formulate tailored instructional programs. In the context of new engineering disciplines, integrating portraits of key subject competence into the classroom represents a noteworthy endeavor to enhance traditional modes of teaching.

### 4.3. An educational model to promote competencies in key disciplines

**Fig 4:**
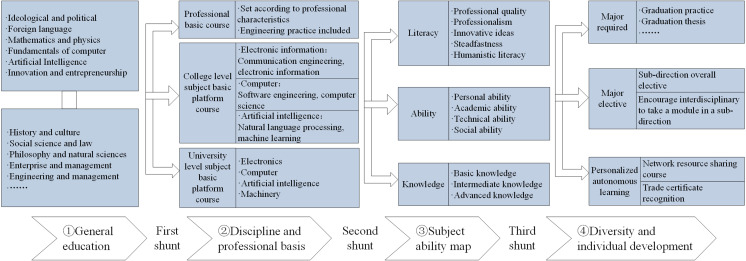
Diagram of the instructional model to develop competencies in key disciplines.

Furthering students’ key disciplinary competence relies on the continual exploration and enhancement of instructional models. Building upon the concept of mapping key disciplinary competencies, a novel instructional model is introduced here to foster the development of student competence in new engineering disciplines by addressing the limitations of the existing instructional model. The current model has been critiqued for its insufficient comprehensive application of multidisciplinary knowledge and its reliance on a singular professional perspective to address application-oriented problems. The new model is illustrated in Fig 4.

Alongside conventional courses such as mathematics, students are enriched in the realm of general education with additional offerings in engineering thinking and project practice, encompassing subjects such as engineering and thinking, system engineering theory and practice, and innovative projects and practice. The transition into the phase focused on the foundation of disciplines and professions initiates the first stream of the model, where students delve into foundational professional courses (with an emphasis on engineering practice), as well as faculty and university platform courses (including courses in artificial intelligence). This stream progresses to foundational disciplinary courses and from engineering and thinking to advanced courses with a focus on engineering thinking and project practice. The second stream unfolds in the transition from disciplinary and specialty foundational courses to discipline competency mapping, emphasizing key LAK competencies in the discipline. The third stream emerges during the shift from discipline competence mapping to diversification and personality development. It underscores the study of both compulsory and optional courses in professional directions, as well as engagement in practical training for small and medium-sized engineering projects (e.g., internships with enterprises and joint training initiatives), and the exploration of personalized independent learning. This model culminates in the realization of the overarching goal—the development of students’ key disciplinary competencies. This instructional model is a viable solution for exploring implementation strategies to foster university students’ key disciplinary competencies within the framework of the NEE initiative.

## 5. Suggested countermeasures for shaping students’ abilities

**Fig 5:**
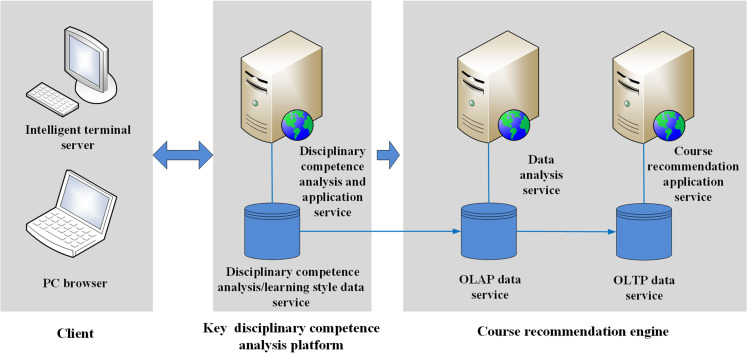
Schematic of the prototype competence-based course recommendation system.

The development of key subject competencies hinges on a judicious formulation of the curriculum. Leveraging the intelligent perception and recommendation engines of the platform for big data disciplinary competence analysis, learners receive course recommendations and learning paths tailored to their individual development based on their current level of competence (i.e., its “state value”) [19]. The prototype system is depicted in Fig 5. For instance, Student A initially obtained a state value for their subject ability and a recommended course learning system outline from the platform. If Student A wishes to embark on the learning path recommended by the platform, they only need to provide their state value for the given subject ability and their learning style (determined by a learning style test) as inputs. The platform’s intelligent recommendation algorithm then generates learning path options aligned with the student’s unique profile.

**Fig 6:**
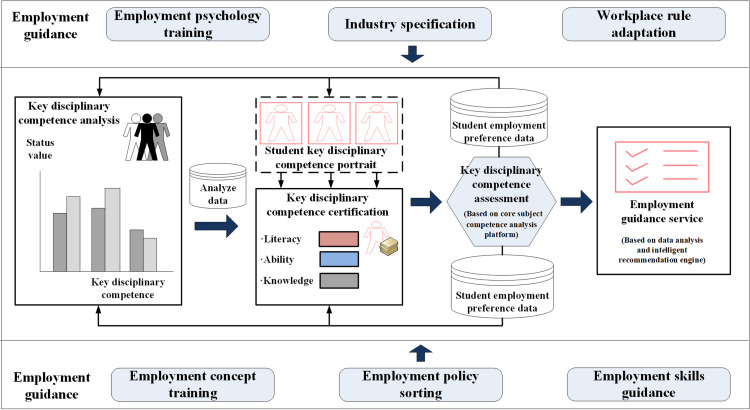
Employment guidance services based on certification of core disciplinary competencies.

In addition to establishing a recommended curriculum to develop key subject competencies, there is also an urgent need to establish career guidance services based on the certification of these competencies [20]. Guided by the digital profile produced by the platform for big data disciplinary competence analysis (i.e., the individual disciplinary competence data structure), a targeted approach has been formulated for training information-related talents in NEE disciplines to meet employment needs based on the certification of key disciplinary competencies. This approach would provide quality assurance in preparatory education for graduates entering the professional sector within the context of the NEE initiative. Competence certification is the cornerstone of this approach for employment guidance. Its significance lies in approaching education from the perspective of students’ disciplinary competence to supply the workforce with high-quality, multidisciplinary human capital tailored to the immediate needs of the job market. The model is illustrated in Fig 6.

## 6. Conclusions

NEE is a testing ground for cultivating the key competencies of high-quality talents. Promoting the development of students’ key competencies is important for evaluating the quality of education. This paper therefore explored values for cultivating key disciplinary competencies among university students against the backdrop of the NEE initiative. Based on the results of a survey on the demand for specific competencies, the LAK model was proposed for the structure of university students’ key disciplinary competencies. Building upon this, course-level implementation research was conducted. First, the key disciplinary competencies were encoded in the field of information in the context of new engineering disciplines to construct a map of course-level competencies, as well as a multi-disciplinary portrait of key disciplinary competencies for university students. Second, using the key disciplinary competence map as a framework, a new instructional model was explored that facilitates students’ competency development. Finally, a course recommendation system that aligned with an employment guidance service was established based on certification for key disciplinary competencies. Future research should build upon the key disciplinary competence analysis platform, with a particular focus on exploring teaching models and furthering research in the field of education quality assessment within the NEE context. The goal of such research should be to better contribute to society by developing high-quality, multidisciplinary human capital in the NEE field.

## Supplementary information

S1 File Data_v1. (XLSX)
